# Supplementary material for: Effects of amino acid value-added urea on rice growth and nitrogen utilization
Source: PLoS One. 2025 Feb 3;20(2):e0310224. doi: 10.1371/journal.pone.0310224 (PMC11790094; doi:10.1371/journal.pone.0310224)
Supplement: Attachment — Submitted filename: Response to Reviewers.doc [file pone.0310224.s003.doc]

Dear Faisal Mamhood,

Thank you very much for giving us an opportunity to revise our manuscript. We appreciate the editor and reviewers very much for their constructive comments and suggestions on our manuscript entitled“Effects of Amino Acid Value-Added Urea on Rice Growth and Nitrogen Utilization”(PONE-D-24-15771)

We have studied the valuable comments from you, the assistant editor and reviewers carefully, and tried our best to revise the manuscript. The point to point responds to the reviewer’s comments are listed as following.

Responds to the reviewer’s comments:

Reviewer 1

**Comment 1:** The abstract should be shortened because it is too lengthy.

**Response:**Thank you for your valuable advice.we have corrected the abstract.Furthermore, we have had the manuscript polished with a professional assistance in writing.

**Comment 2:** In the introduction part: add more data about the literature.

**Response:**Thank you for your valuable advice.The existing literature extensively covers the physiological effects of amino acids on crops and the combined effects of amino acids and nitrogen fertilizers. However, there is a scarcity of research on the mechanism of value-added urea resulting from the fusion of amino acids and urea. This lack of data hinders a comprehensive understanding of the role and mechanism of value-added urea. Our introduction section follows a logical progression: exploring fertilizer innovation due to adverse reactions from traditional nitrogen fertilizer application, emphasizing the impact of nitrogen forms on rice growth and nitrogen absorption, highlighting the novel aspect of adding different amounts of amino acid value-added urea to improve rice nitrogen utilization, and ultimately aiming to provide insights for optimizing rice yield composition and effectively utilizing amino acid value-added fertilizers.

**Comment 3:** Make appropriate headings and heading numbers for the manuscript according to the journal format.

**Response:**Thank you for your valuable advice.The article has been modified to meet the requirements of the PLOS ONE's style in terms of paper format.

**Comment 4:** Results and discussion (Improve discussion part and Provide literature data).

**Response:**Thank you for your valuable advice.The article has been rewritten in the Results and Discussion section.

**Comment 5:** Conclusion (Rewrite as it is not up to the mark author should be more focused on current study results).

**Response:**Thank you for your valuable advice.The article has been rewritten and the conclusion section has been written.

**Comment 6:** The language of this manuscript is poor. I suggest the authors get help from a native English-speaking person.

**Response:**Thank you very much to point out the sentence structure and grammatical issues in our manuscript. According to the comments from you and the editors, we polished the manuscript with a professional assistance in writing, conscientiously.

**Comment 7:** Grammatical error.

**Response:**Thank you very much to point out the sentence structure and grammatical issues in our manuscript. According to the comments from you and the editors, we polished the manuscript with a professional assistance in writing, conscientiously.

**Comment 8:** Require Reference justification and alignment.

**Response:**Thank you for your valuable advice.The reference section of the article has been revised and aligned

Reviewer 2

**Comment 1:** Very poor english writeup, difficult to understand.

**Response:**Thank you very much to point out the sentence structure and grammatical issues in our manuscript. According to the comments from you and the editors, we polished the manuscript with a professional assistance in writing, conscientiously.

**Comment 2:** Treatments are not understandable.

**Response:**Thank you for your valuable advice.The article has redefined the Treatments.

**Comment 3:** Data is not sufficient, there must be some growth and yield attributes as well.

**Response:**Thank you for your valuable advice.Due to confidentiality agreements and other reasons, other data has not been made public yet, so it is not convenient to add it to the article. Please understand

**Comment 4:** Result interpretation is also not well.

**Response:**Thank you for your valuable advice.The article has been rewritten in the Results and Discussion section.

**Comment 5:** Discussion need to be improved according to latest review.

**Response:**Thank you for your valuable advice.The article has been rewritten in the Results and Discussion section.

Once again, thank you very much for your constructive comments and suggestions which would help us both in English and in depth to improve the quality of the paper.

Kind regards.

Lin CHENG

E-mail:1550708563@qq.com
